# Confinement-Driven Segregation Enables Glassy Polymer Hybrid Materials Featuring Disordered Hyperuniformity and Integrated Self-Healing

**DOI:** 10.1021/acsmaterialslett.5c00878

**Published:** 2025-07-12

**Authors:** Hanshu Wu, Yuqi Zhao, Jirameth Tarnsangpradit, Ted Autore, Jaepil Jeong, Krzysztof Matyjaszewski, Michael R. Bockstaller

**Affiliations:** † Department of Chemistry, Carnegie Mellon University, 4400 Fifth Avenue, Pittsburgh, Pennsylvania 15213, United States; ‡ Department of Materials Science & Engineering, Carnegie Mellon University, 5000 Forbes Avenue, Pittsburgh, Pennsylvania 15213, United States

## Abstract

The blending of glassy copolymer-brush modified colloids with a viscoelastic linear copolymer featuring intrinsic self-healing enabled disordered hyperuniform hybrid materials that combined mechanical robustness with structural color, processability, environmental stability, and the ability to recover structure and properties after incurring physical damage via ‘integrated self-healing’. Symmetric linear n-butyl acrylate/methyl methacrylate (BA/MMA) were co-assembled with asymmetric glassy BA/MMA statistical copolymer brush (silica) particles. ‘Confinement-driven segregation’ resulted in a microphase-separated morphology in which the linear copolymer resided within the interstitial regions of a rigid (∼1 GPa) copolymer brush particle template with disordered hyperuniform microstructure. Diffusion of the self-healing copolymer additive into damage regions drove the recovery after damage, along with the restoration of structural color due to the materials hyperuniform microstructure. The synergistic action of intrinsic and extrinsic healing mechanisms could provide a versatile platform for the bottom-up fabrication of multifunctional hybrid materials with increased damage resistance and functional longevity.

## Graphical abstract

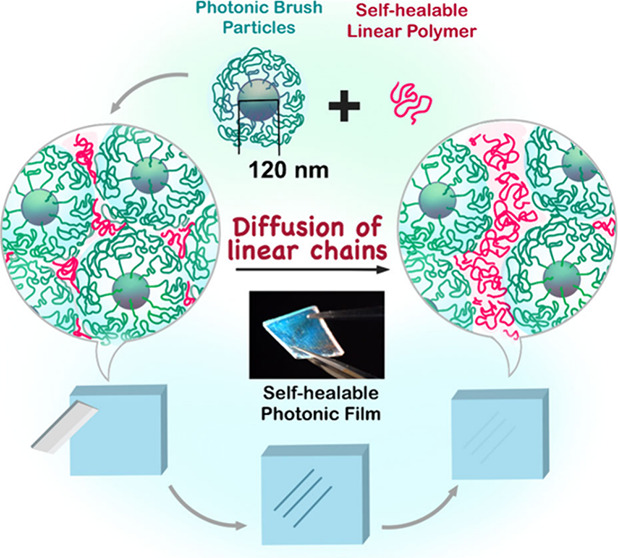

The ability of biological tissues to self-repair and resume functional performance after incurring structural damage has inspired research to develop engineering polymers with ‘self-heal ability’. The first reports of ‘healing’ in polymer materials date to 1978, when structural recovery of filled elastomers (used as solid fuel propellants) was reported.^1^ Since then, interest in endowing polymers with ‘self-healing’ capability has rapidly accelerated due to the growing relevance of the longevity and sustainability of polymer products and devices.^2–9^ The mechanisms of self-repair processes in polymeric materials are complex and dependent on the material system. Recovery in homogeneous thermoplastics is commonly accomplished in the rubbery regime when chain dynamics is fast enough to facilitate welding and interdiffusion across interfaces.^4,6–10^ Thus, self-repair on practical time scales typically requires temperatures in excess of a polymer’s glass transition temperature, *T*_g_. This has rendered the realization of rigid polymers that ‘self-heal’ on practical time scales and temperatures a challenge.

To afford the ‘self-healing’ of high modulus polymers (here defined as polymers with elastic modulus *E* ≳1 GPa), both extrinsic and intrinsic strategies have been developed with the goal to decouple the dynamical properties of the bulk and damage regions. Extrinsic ‘self-healing’ involves the dispersion of low molecular healing agents, e.g., through vascularization or encapsulation.^11,12^ This approach, however, is limited by the lack of scalability and processability of the resulting materials. In contrast, intrinsic ‘self-healing’ is accomplished by the integration of dynamic (i.e., reversible) covalent or noncovalent bond chemistries.^13–19^ Damage causes localized breaking of the dynamic bond linkages. The resulting reduction in bond density accelerates the local dynamics of chains in the damaged region and thus promotes the reconstitution of bonds across the fracture surface. Unfortunately, suitable chemistries to realize reversible bond formation are often complex and expensive or render materials sensitive to environmental conditions such as temperature and humidity.^14,20–27^

The respective advantages and disadvantages of extrinsic and intrinsic healing mechanisms motivated us to consider high-strength hybrid materials with *integrated* self-healing ability, i.e., materials in which extrinsic *and* intrinsic mechanisms synergistically combine to enable high modulus/strength materials with low-temperature structure and property reconstitution. The approach builds on the recent discovery of self-healing in symmetric ‘key-and-lock’ poly­(*n*-butyl acrylate/methyl methacrylate) (BA/MMA) statistical copolymers and utilizes the concept of ‘confinement-driven segregation’ that enables the localization of a filler within the interstitial regions of a template formed by polymer-grafted nanoparticles (aka ‘brush particles’, PGN).^10,28–30^ The coassembly of MMA-rich statistical BA/MMA copolymer PGNs and linear statistical BA/MMA copolymers with symmetric composition thus resulted in a microstructured hybrid material in which PGNs formed a rigid template (with elastic modulus *E* ∼ 1 GPa) that hosted mobile ‘key-and-lock’ copolymers within its interstitial volume. Diffusion of the self-healing linear copolymer within the interstitial regions instigated structure recovery, while the structure and dynamical properties of the template imparted additional functionality, such as structural color and shape-memory behavior. ‘Integrated self-healing’ based on brush particle templates thus presents a versatile platform approach to enable high modulus, multifunctional hybrid materials that cannot be realized using existing methods that rely on the blending of self-healing polymers or the dispersion of low-molecular healing agents within self-healing polymer matrices.^31,32^ The concept is illustrated in Figure 1.

**Figure 1:**
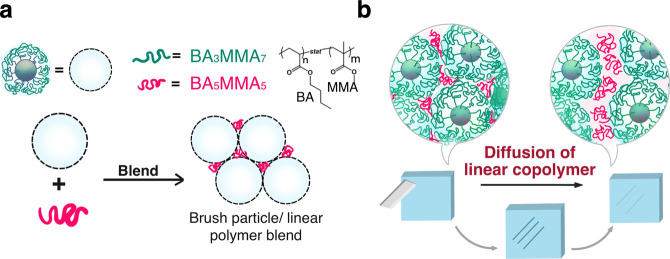
Illustration of ‘integrated self-healing’ in statistical BA/MMA copolymer/brush particle blend systems (subscripts denote the stochiometric composition of the respective copolymer). (a) 'Microsegregated' morphology featuring linear copolymer fillers concentrated within the interstitial sites of the glassy/rigid copolymer-brush particle template. (b) Proposed self-healing mechanism of the blended system via transport of self-healing linear copolymer through interstitial regions to damage the site.

Surface-initiated atom transfer radical polymerization (SI-ATRP) was utilized to synthesize poly­(BA-*stat*-MMA)-grafted silica (*d* = 113.2 ± 12 nm) brush particles with a 68.9 mol % MMA content via a ‘grafting-from’ approach.^33,34^ Linear poly­(BA-*stat*-MMA) copolymers, with 51.1 mol % MMA and shorter chain lengths compared to the template, were synthesized by the supplemental activator and reducing agent (SARA)-ATRP. The lower degree of polymerization of the linear copolymer was intended to increase its miscibility within the brush particle template and to prevent macrophase separation.^35,36^ A brush molar composition of BA:MMA = 3:7 was chosen to enable a high modulus template, while the approximately equimolar composition of the linear copolymer was previously shown to impart self-healing ability. All reactions were restricted to the low conversion limit to obtain statistical sequence.^29,30,34^ The particle brush systems will be abbreviated as SiO_2_-B_X_M_Y_, where SiO_2_ is the nanoparticle composition; X and Y are the molar compositions of B (BA) and M (MMA), respectively. For linear analogs, the prefix ‘SiO_2_’ is omitted. The blended systems will be abbreviated as Blend-Z%, where Z% is the volume fraction of linear copolymer in the blend system. The relevant molecular and compositional details are presented in Table 1.

**Table 1:** Characteristics of the SiO_2_-g-P­(BA-*stat*-MMA) Copolymer Brush Particle, BA-*stat*-MMA Copolymer, and Corresponding Blend Systems

Entry^a^	***x***_BA_ (mol %)^b^	***x***_MMA_ (mol %)^b^	***M***_n_ ^c^	***M***_w_/***M***_n_^c^	***f***_SiO2_ (%)^d^	(nm^–2^)^e^	***T***_g1_ (°C)^f^	***T***_g1_ Range (°C)^f^	***T***_g2_ (°C)^f^	***T***_g2_ Range (°C)^f^
**SiO**_**2**_ **–B**_**3**_ **M**_**7**_	31.1	68.9	111,840	1.23	22.33	0.81	43	[28, 53]	—	—
**B**_**5**_ **M**_**5**_	48.9	51.1	30,770	1.24	—	—	—	—	9	[−3, 19]
**Blend-10%**	—	—	—	—	—	—	44	[24, 58]	8	[0, 18]
**Blend-20%**	—	—	—	—	—	—	44	[22, 53]	7	[0, 16]
**Blend-30%**	—	—	—	—	—	—	45	[19, 60]	9	[−2, 18]
**Blend-50%**	—	—	—	—	—	—	45	[23, 61]	9	[−5, 16]
**B**_**3**_ **M**_**7**_	31.1	69.9	33,300	1.27	—	—	39	[24, 50]	—	—

a Reaction conditions are listed in the Supporting Information.

b Determined by ^1^H NMR. The NMR results are shown in Figure S1.

c Determined by SEC.

d Determined by thermogravimetric analysis (TGA).

e Calculated by eq 1.

f The glass transition temperatures are determined by DSC. Silica nanoparticle diameter *d* = 113.2 ± 12 nm.

The morphology of the SiO_2_-B_3_M_7_ and SiO_2_-B_3_M_7_/B_5_M_5_ blend systems was studied by transmission electron microscopy (TEM) and small angle neutron scattering (SANS) to evaluate microstructure uniformity. Electron micrographs such as Figure 2(a, b) as well as Figure S2 revealed a uniform microstructure for the pristine sample, Blend-10%, Blend-20% and Blend-30% – no large-scale phase separation was observed. Particle center-to-center distances *D* were determined from the average distance between the centers of adjacent Voronoi cells after the tessellation of images (representative distributions of interparticle distances are shown in the insets of Figure 2(a, b). *D* increased with the fraction of linear copolymer as 215 ± 33 nm (SiO_2_-B_3_M_7_), 214 ± 39 nm (Blend-10%), 219 ± 25 nm (Blend-20%), and 221 ± 34 nm (Blend-30%). The fractional increase of interparticle distances *D*_ϕ_/*D*_0_ was less than the corresponding values for uniformly diluted structures (i.e., with the linear filler uniformly distributed within the film), for which *D*_ϕ_/*D*_0_ ≙ (1 + ϕ)^1/3^, with ϕ representing the linear copolymer volume fraction, would be expected. This indicated that the linear copolymer was not uniformly dissolved within the brush particle templates but rather concentrated within the interstitial regions of the brush particle assembly (Figure 1a). Similar observations were first reported by Schmitt et al., who proposed that the relaxation of stretched brush chains within the interstitial regions of the assembly drives the local segregation of polymer fillers.^37^ Consistent with this hypothesis was the dependence of *D* on the amount of added B_5_M_5_. Whereas for ϕ = 0, 0.1, and 0.2, *D* remained about constant (within the experimental certainty), a pronounced increase was observed for Blend-30% that could be rationalized as the volume fraction of linear copolymer starting to exceed the volume of interstitial regions. Thus, we hypothesize that in the Blend-30% system filling of interstitial volume was complete and a fraction of the linear copolymer contributed to the uniform swelling of the brush particle template. Further increase of linear copolymer content to ϕ = 0.5 resulted in the formation of a macrophase-separated structure (Figure S3). We note that a reference B_3_M_7_/B_5_M_5_ blend of linear copolymers was fully miscible at all tested compositions (ϕ = 0.1, 0.2, 0,3) as indicated by singular glass transition temperatures (Figure S4). This supported that those energetic contributions unique to brush particle assembly structures (such as the relief of packing frustrations within interstitial regions) drive the segregation of linear copolymer, even in otherwise miscible blend compositions.

**Figure 2:**
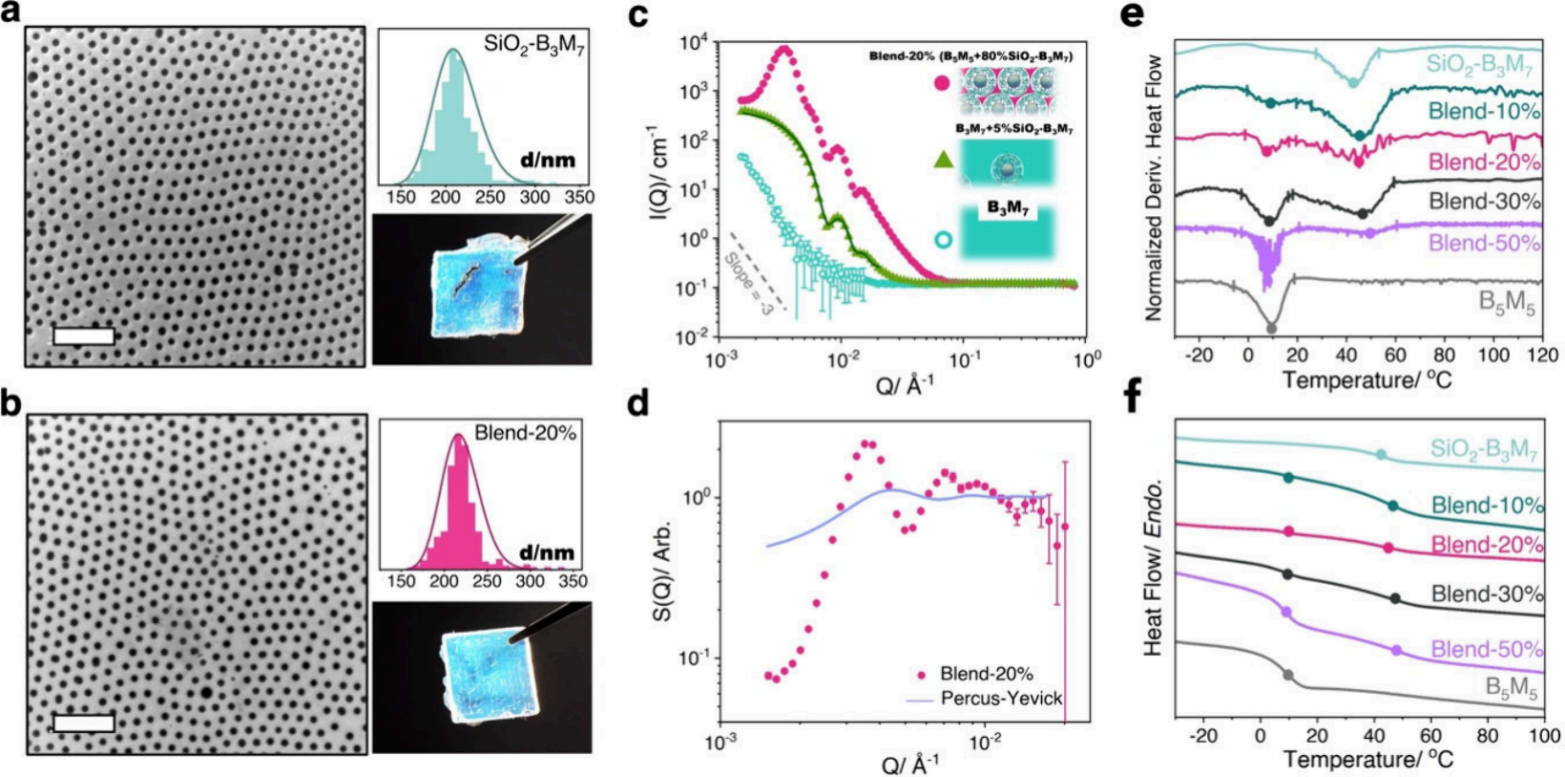
Structural analysis of SiO_2_-B_3_M_7_ and Blend-20%. (a, b) Bright field TEM image, interparticle distance distribution, and photograph of SiO_2_-B_3_M_7_ and Blend-20%. Scale bars are 1 μm. (c) SANS results of Blend-20% (red filled circles), B_3_M_7_ (blue open circles), and the reference B_3_M_7_ + 5% SiO_2_-B_3_M_7_ (green triangles)_._ Green line in B_3_M_7_ + 5 wt % SiO_2_-B_3_M_7_ represents the fit corresponding to a disperse sphere form factor, yielding a radius of 61.3 ± 8.2 nm for the SiO_2_ particle size. (d) Structure factor results of Blend-20% along with the Percus–Yevick approximation for corresponding spheres with radius 61.3 ± 8.2 nm and volume fraction 0.12 (corresponding to the volume fraction of silica in Blend-20%). (e) Normalized derivative heat flow curves and (f) heat flow curves. The *T*_g_ values are highlighted with solid points, and the *T*_g_ onsets and offsets are marked as solid lines in the figure. Sample identification is as follows: Light blue line: SiO_2_-B_3_M_7_; dark cyan line: Blend-10%; magenta line: Blend-20%; black line: Blend-30%; purple line: Blend-50%; gray line: B_5_M_5_. All curves were recorded during the 3rd heating/cooling run at a heating rate of 20 °C min^–1^.

Small angle neutron scattering (SANS) experiments were performed to reveal the structure of bulk materials and the origin of structural color that was observed in all systems (see insets in Figure 2a and 2b). Here, we note that the structure of bulk materials cannot be inferred from electron imaging of monolayers due to the effect of substrate interactions and spatial confinement on the film microstructure. Figure 2c displays the (absolute) scattering intensity *I*(*Q*) of Blend-20% (red full circles) as well as the respective reference systems B_3_M_7_ + 5% SiO_2_-B_3_M_7_ (green triangles) and B_3_M_7_ (blue circles). To determine the structure factor *S*(*Q*) of the particle distribution in the pristine and blended systems, the form factor *P*(*Q*) associated with the scattering of individual brush particles was determined first. Dilute (1% and 5%) dispersions of SiO_2_-B_3_M_7_ within a linear B_3_M_7_ polymer matrix were prepared by solution casting. Structural uniformity was concluded from TEM as well as the analog *Q*-dependence of the scattering intensity of the 1% and 5% solution (not shown here). The scattering length density (SLD) values of the particle cores and polymer matrix were calculated to be SLD_SiO2_ = 3.47 × 10^10^ cm^–2^ and SLD_B3M7_ = 9.4 × 10^9^ cm^–2^, respectively. This allowed us to quantitatively represent the scattering intensity with the form factor *P*(*Q*) of disperse spheres with a radius of 61.3 ± 8.2 nm, in good agreement with TEM results. Figure 2d represents the calculated structure factor *S*(*Q*) = *I*(*Q*)/*P*(*Q*) for the Blend-20% system (red circles) where SLD_B5M5_ = 8.6 × 10^9^ cm^–2^ ≈ SLD_B3M7_ was assumed. Several pertinent conclusions can be drawn based on the experimental structure factor. First, the distinctive peak at Q* = 3.3 × 10^–3^ Å^–1^ reveals the existence of a characteristic spacing 2π/Q* = 178.9 nm. Similar calculations did yield 178.9, 185.2, and 191.9 nm for Blend-0%, Blend-10%, and Blend-30%, respectively, thus confirming the trend from the TEM monolayer analysis (note that absolute values differ since characteristic spacings in *S*(*Q*) do not necessarily correspond to interparticle distances that are discerned from electron images). Second, several broad peaks in *S*(*Q*) suggest a regular structure that is, however, not crystalline. The light-purple line in Figure 2d depicts the calculated Percus–Yevick structure factor of a hard sphere fluid that matches the characteristics and concentration of silica particles in the polymer matrix.^38^ The increase of amplitude and the number of peaks of the experimental *S*(*Q*) confirm a more regular structure as compared to the hard-sphere fluid analog. Yet, peak positions could not be attributed to a crystal structure (such as face-centered (FCC), body-centered cubic (BCC) or hexagonal close-packed (HCP), Figure S5). Further, the suppression of the amplitude of higher-order peaks did not indicate long-range periodicity that would be expected for a crystal-like order of particle positions as was observed in artificial opal-type colloidal crystals.^39^ We, therefore, propose that a more appropriate classification of the structure of brush particle solids tested in our experiments was as ‘disordered hyperuniform’ packings. Disordered hyperuniform materials feature short-range order and the suppression of long-distance fluctuations, thus combining structural features common to both amorphous and crystalline materials.^40^ To support this proposition, we determined the apparent degree of hyperuniformity following Torquato and Stillinger as η = *S*(*Q*_min_)/*S*(Q*) where *S*(*Q*_min_) is the value of the structure factor at the smallest measured scattering vector and *S*(Q*) is its value at the first peak position.^40^ For the Blend-20% system (Figure 2c) η = 0.036 was found, close to the threshold value η_crit_ = *S*(0)/*S*(Q*) = 10^–3^ that has been proposed by Torquato et al. as an upper bound for hyperuniform materials (note that, since η is determined at finite *Q*_min_ rather than at *Q* = 0, the experimental values represent the ‘apparent’ degree of hyperuniformity).^40^ The observation of near hyperuniformity supports recent molecular dynamics simulations by Chremos and Douglas, who suggested brush particles (and other crowded polymeric systems such as stars or bottlebrush polymers) to be capable of forming hyperuniform states.^41^ We thus propose that structural color in the studied systems was due to uniform short-range order rather than structural periodicity, as is common for ‘conventional’ hard-sphere-type colloidal crystal materials.^42–44^ We note that the apparent degree of hyperuniformity correlated with the fraction of added linear copolymer as η = 0.032 (pristine SiO_2_-B_3_M_7_), 0.03 (Blend-10%), 0.036 (Blend-20%), and 0.072 (Blend-30%), respectively. We hypothesize that the decrease of uniformity (indicated by the increasing value of η) with linear copolymer addition was due to the increased number of degrees of freedom of blended systems that promote compositional fluctuations during assembly formation. However, in all systems, uniformity was found to be sufficient to induce structural color of the hybrid materials (Figure S6).

Insight into the distribution of B_5_M_5_ filler within brush particle solids was derived from differential scanning calorimetry (DSC) traces. Heat flow curves (Figure 2e) and derivative heat flow curves (Figure 2f) revealed two distinct glass transitions of blended systems that can be attributed to SiO_2_-B_3_M_7_ (*T*_g_ ≈ 44 °C) as well as B_5_M_5_ (*T*_g_ ≈ 9 °C). This further supported a 'microsegregated' morphology, in which the linear copolymer filler concentrates within the interstitial regions of the hybrid in phase-pure form, thus causing a glass transition like the pristine (pure) B_5_M_5_.

To assess the mechanical properties of SiO_2_-B_3_M_7_/B_5_M_5_ blend systems, uniaxial tensile testing (23 °C, 0.05 s^–1^) was performed to determine the Young’s modulus (*E*) and toughness (*U*) of materials. Films (15 × 5 × 0.2 mm^3^) were prepared by casting from 5% THF solution and subsequent vacuum annealing at 100 °C. Measurements were performed using a TA RSA-G2. Figure 3 displays representative stress–strain curves (Figure 3a) and mechanical properties (Figure 3b) that were determined by evaluating the slope within the elastic regime (Young’s modulus, blue bars) as well as the area under the stress–strain curve (toughness, red bars). The figure revealed two pertinent trends: First, in the limit of small amounts of added B_5_M_5_ , *E* was about independent of the fraction of added B_5_M_5_ and approximately equal to the pristine copolymer brush particle template (SiO_2_-B_3_M_7_: 0.92 GPa, Blend-10%: 0.83 GPa; Blend-20%: 0.86 GPa). Since the measured elastic modulus of brush particle solids is determined by short-ranged dispersion interactions between segments in adjacent brush layers, this trend confirmed that segregation within interstitial regions did not fundamentally alter interactions within the template.^45^ Accordingly, increasing the filling fraction of B_5_M_5_ beyond ϕ_crit_ ≤ 0.3 (corresponding to the capacity limit of interstitial regions) resulted in the rapid decrease of *E*, indicating a dilution of brush–brush contacts by the softer B_5_M_5_ (Blend-30%: 0.63 GPa; Blend-50%: 0.18 GPa). This interpretation is supported by the comparison of *E* with the predicted effective medium values for composite materials that were calculated using the Voigt (*E*_U_ = Σ*E*_*i*_ϕ_*i*_) and Reuss (*E*_L_ = (Σϕ_*i*_/*E*_*i*_)^−1^) averages, that were assumed as approximations to the upper and lower bound, respectively.^46^ Whereas Blend-20% exceeded the expectation value even of the upper bound (thus confirming the absence of any dilution effect on interactions due to B_5_M_5_ addition), the value of Blend-50% was well represented by the lower bound (consistent with continuity of the soft B_5_M_5_ phase due to phase separation, Figure S3). Concurrently, the toughness, *U*, increased by about one order-of-magnitude upon addition of linear copolymer (SiO_2_-B_3_M_7_: 0.17 MJ/m^3^; Blend-10%: 3.29 MJ/m^3^; Blend-20%: 7.38 MJ/m^3^) before decreasing in the systems Blend-30% (3.66 MJ/m^3^) and Blend-50% (4.55 MJ/m^3^). Since toughness is related to the energy dissipation during plastic deformation and fracture, this trend should be due to the cumulative impact of B_5_M_5_ addition on interactions, dynamics, and entanglement of blended systems. While difficult to interpret, the results demonstrate that the toughness of brush particle templates can be selectively enhanced by tailored addition of linear (co)­polymer without detriment to the materials modulus.

**Figure 3:**
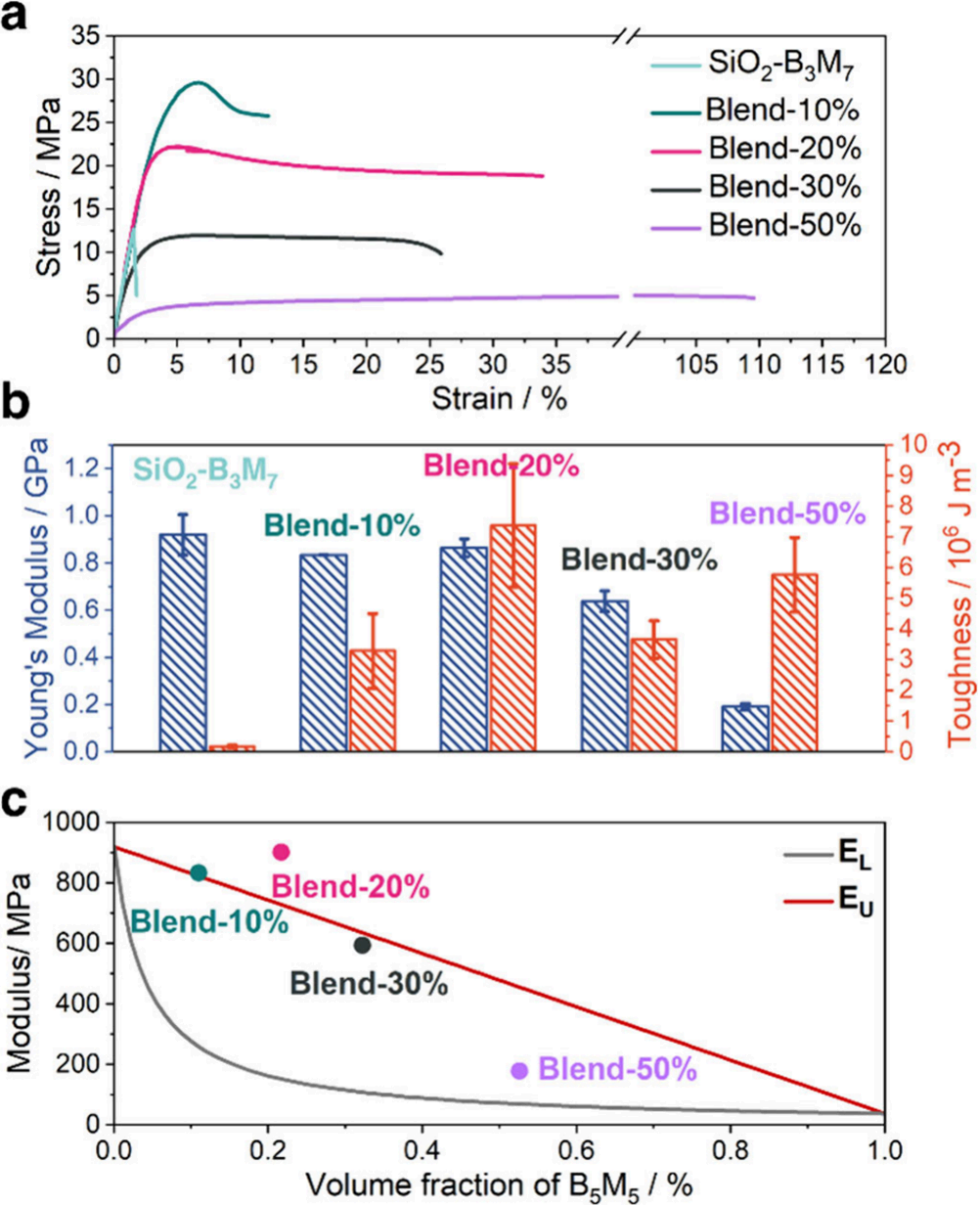
Comparison of mechanical properties of SiO_2_-B_3_M_7_/B_5_M_5_ films. (a) Representative stress–strain curves of SiO_2_-B_3_M_7,_ Blend-10%, Blend-20% and Blend-30% measured by uniaxial tensile testing (23 °C, 0.05 s^-1^). (b) Comparison of Young’s modulus (blue bars) and toughness (red bars) of pristine and blend systems. (c) Comparison of Young’s modulus of blend systems with the Voigt modulus (upper bound, red line) and the Reuss modulus (lower bound, black line). *E* > *E*_U_ at ϕ = 0.2 confirms that for Blend-20% the soft B_5_M_5_ did not reduce brush interactions, consistent with interstitial segregation (see text for detail; Video S1 and Video S2 compare the impact of toughness on the flex deformation of pristine and Blend-20%).

To evaluate the ability of B_5_M_5_ addition to instigate self-healing in SiO_2_-B_3_M_7_/B_5_M_5_ blend systems, films of SiO_2_-B_3_M_7_ and the respective blend systems were subjected to scratch healing tests. Films of 0.2 mm thickness were indented to result in scratches of about a 100 μm depth using a razor blade. The scratched films were subsequently annealed at 70 °C for a defined time before being cooled to room temperature, and the film topography was analyzed using a stereomicroscope. Figure 4 illustrates the process and shows optical images revealing near-complete scratch-healing in all blend systems after 0.5 h of annealing. The scratch-healing in the blend system could be attributed to the linear copolymer rather than the brush particles, as the pristine SiO_2_-B_3_M_7_ featured a negligible restoration. The depth of the scratch was found to have negligible influence on the healing efficiency across the 50–100 μm range (Figure S7). Our interpretation is based on work by Bilchak et al., who reported interstitial regions in brush particle assembly structures to feature ‘connectivity’, thus enabling transport across brush particle films.^47^ We therefore hypothesized that the healing could be facilitated by the diffusion of the B_5_M_5_ copolymer additive into the damaged region. To test this hypothesis, cut-and-adhere testing on Blend-20% was performed following previously reported procedures (Figure S8).^29^ After recovery at 70 °C for 24 h, the Young’s modulus of the healed sample only reached 0.13 GPa, which is 15% of the modulus of the pristine sample and close to the pristine B_5_M_5_ (0.03 GPa). This indicated that the welding of the cuts involved the concentration of B_5_M_5_ at the interface, thus lowering the modulus of the material after recovery. Scratch-healing could also be achieved by using a hairdryer after 3 min of modest heating (Figure S9). We note that in the Blend-30% system (and similarly Blend-50%, not shown here), scratch-healing remained incomplete after 5 h of annealing. This could be explained by the softer characteristics (Figure 3b) of films that resulted in a more pronounced nonrestorable plastic deformation of the bulk film surface during the scratching process.

**Figure 4:**
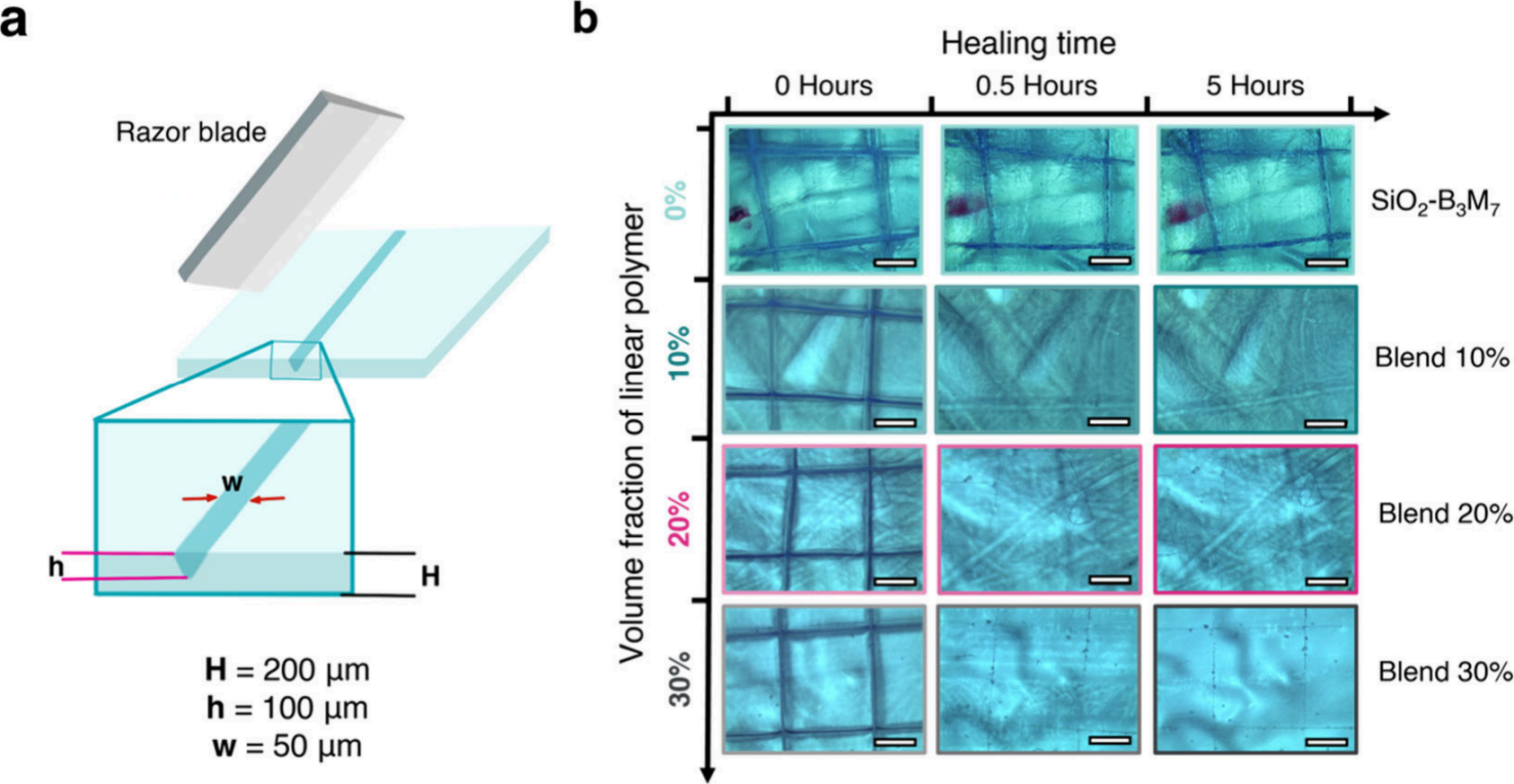
Structure recovery of SiO_2_-B_3_M_7_/B_5_M_5_ films after scratch deformation. (a) Illustration showing the experimental process. (b) Stereomicroscopic images of the healing process for blends with different volume fractions of the linear B_5_M_5_ copolymer. Scratch healing was performed at 70 °C. Scale bars are 500 μm.

Interestingly, the SiO_2_-B_3_M_7_/B_5_M_5_ hybrid materials featured not only self-healing but also shape-memory behavior. The shape memory capability of the Blend-20% was evaluated using ‘origami’-inspired 3D structure transitions such as the folding of a flower-shaped structure as seen in Figure 5. A ‘flower’ shape was cut from a film of Blend-20% with 0.1 mm thickness and heat-pressed into a permanent 3D flower shape at *T* = 70 °C. The ‘flower’ was then pressed into a 2D (planar) shape at 70 °C and subsequently quenched to room temperature. Folding was then induced by heating to 70 °C, which caused the film to restore its 3D ‘flower’ shape within 30 s of heating (Video S3). The observation of dual self-heal/shape-memory behavior resembles earlier reports for pristine SiO_2_-B_5_M_5_ brush particle solids.^34^ In this previous work, it was shown that the slow displacement kinetics of brush particles imparts a ‘double network-type’ character to brush particle solids, whereby the jamming of (effectively) immobile particle cores provides a mechanism for strain energy storage that drives shape restoration upon heating of the materials to temperatures high enough so that the dynamics of brush chains provides a sufficiently mobile environment for shape restoration on experimental time scales.^34^ We hypothesize that a similar mechanism is responsible for the shape-memory behavior of SiO_2_-B_3_M_7_/B_5_M_5_, despite its significantly higher elastic modulus of ∼0.9 GPa (as compared to the 0.15 GPa of the previously tested SiO_2_-B_5_M_5_). The blend film also possessed good reprocessability (Figure S10), which was demonstrated by cut-and-recycle processing of Blend-20% bulk films. After being cut into pieces with a razor blade, the films could be reformed via hot-pressing at 70 °C for 5 min. The structural color was retained after reprocessing, indicating that the structure uniformity of brush particles was not destroyed during the recycling process. This is rather atypical for colloidal-crystal type materials, in which optical properties sensitively depend on grain size and orientation, which are subject to change during processing. We attribute this feature to the disordered hyperuniform rather than crystalline characteristics of SiO_2_-B_3_M_7_/B_5_M_5_ blends. The shape memory effect and viable formability should open diverse application opportunities of the blend systems in areas such as photonic films and coatings.^48–50^

**Figure 5:**
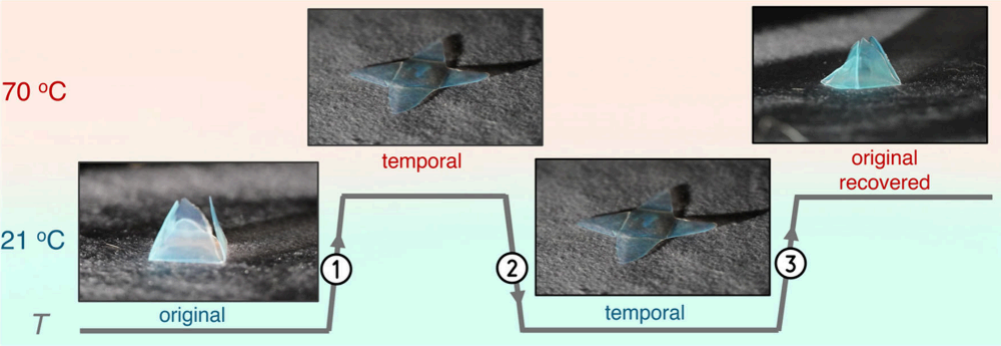
Illustration of the shape memory capability of the SiO_2_-B_3_M_7_/B_5_M_5_ films. Shape memory action of Blend-20% bulk film. A cut film was folded into a 3D ‘flower’ shape at 70 °C (original shape). Step 1: Unfolding of film into a temporal flat shape using a hot press (70 °C, 100 psi, 2 min). Step 2: Quenching of flattened film to 21 °C. Step 3: Recovery of folded (original) shape after 30 s at 70 °C on a hot plate (see also Video S3)

In conclusion, 'confinement-driven segregation’ spurs the localization of linear copolymers with intrinsic self-heal ability within the interstitial regions of a copolymer brush particle template featuring a higher concentration of glassy components. The synergistic combination of the mobile self-healing copolymer filler and the structural rigidity of the brush particle template enables hybrid materials featuring a Young’s modulus of 0.9 GPa as well as dual self-healing and shape-memory ability that can be (re)­processed by molding or casting. To further exploit this new class of copolymer brush hybrid materials, future research will need to elucidate the role of copolymer brush architecture, compositional asymmetry, degree of polymerization, and sequence on the distribution of linear copolymer and the respective impact on modulus and toughness of materials. An open question also concerns the kinetics of re-establishing the equilibrium microstructure of the pristine blend system after healing, as this is expected to determine the recovery rate and final state of mechanical properties. An intriguing observation is the high level of disordered hyperuniformity of self-assembled brush particle solids, as this could provide a path toward hybrid materials with more robust and isotropic structural color that could find use in, for example, scratch-resistant photonic coatings.

## Supplementary Material









## Notes

Details of synthesis procedures, characterization procedures (DSC, tensile test, DMA, self-healing test, adhesive tests), derivative heat flow curves, strain–stress, and self-healing data. (PDF)
Bending process of bulk film of SiO_2_-B_3_M_7_. (MOV)
Bending process of bulk film of Blend-20%. (MOV)
Shape memory process for Blend-20% bulk film. (MOV)
