# Supplementary material for: Confinement-Driven Segregation Enables Glassy Polymer Hybrid Materials Featuring Disordered Hyperuniformity and Integrated Self-Healing
Source: ACS Mater Lett. 2025 Jul 12;7(8):2902–9. doi: 10.1021/acsmaterialslett.5c00878 (PMC12326392; doi:10.1021/acsmaterialslett.5c00878)
Supplement: Supplementary file 1 [file tz5c00878_si_001.pdf]

Supporting Information

Confinement-Driven Segregation Enables Glassy Polymer Hybrid Materials Featuring Disordered Hyperuniformity and Integrated Self-Healing

Hanshu Wu, Yuqi Zhao, Jirameth Tarnsangpradit, Ted Autore , Jaepil Jeong, Krzysztof Matyjaszewski, Michael R. Bockstaller**

Supporting text

Methods

Materials. Cu^0 (wire, diameter 0.25 mm, 99.9+%, Aldrich) was washed with methanol/ HCl and then with fresh methanol shortly before use. SiO_2-Br initiator was prepared as reported[44]. Ethyl α-bromoisobutyrate (EBiB, 98%, Sigma Aldrich), anisole (99%, Aldrich), tetrahydrofuran (THF, 99%, VWR), methanol (99%, VWR), tris(2-dimethylaminoethyl) amine (Me_6TREN, 99%, Alfa), copper(II) bromide ($CuBr_2$, 99%, Aldrich), N,N-dimethylformamide (DMF, certified, Fisher Chemical), Hydrofluoric acid (HF, 50 vol%, Acros Organics). Monomers: n-butyl acrylate (BA, 99%, Aldrich), methyl methacrylate (MMA, 99%, Aldrich) were purified by passing through a column filled with basic alumina to remove the inhibitor. Alumina (basic, Super I, 50-200 μ m, Sorbtech).

Synthesis of linear copolymer and particle brushes. Initiator (EBiB/SiO_2-Br), monomer: BA and MMA, solvents (anisole), $CuBr_2$, and Me_6TREN, molar ratios shown in supporting information, were mixed thoroughly in a sealed Schlenk flask, followed by the addition of a piece of Cu^0 wire (length ~ 1.5 cm) and degassing by bubbling with nitrogen. The flask was immediately put into an oil bath set at 60 ℃. The conversion was monitored and controlled under 10% by 1H-NMR. The final products were precipitated in cold methanol and then dissolved and stored in THF.

Molar ratios for synthesis of SiO_2-B_3M_7, SiO_2-B_5M_5, B_5M_5 and B_3M_7 via SARA ATRP.

For SiO_2-B_3M_7, Initiator (SiO_2-Br, 0.6g), monomer: MMA (7.52 g, 8.0 mL) and BA (9.79 g, 11 mL), solvents (anisole 10 mL, DMF 2mL), $CuBr_2$ (0.01 g in 2 mL DMF), and Me_6TREN (0.02 mL).

For SiO_2-B_5M_5, Initiator (SiO_2-Br, 0.6g), monomer: MMA (3.76 g, 4.0 mL) and BA (12.46 g, 14 mL), solvents (anisole 8 mL, DMF 2 mL), $CuBr_2$ (0.01 g in 2 mL DMF), and Me_6TREN (0.02 mL).

For B_5M_5, Initiator (eBiB, 0.005 mL), monomer: MMA (3.76 g, 4.0 mL) and BA (12.46 g, 14 mL), solvents (anisole 8 mL, DMF 2 mL), $CuBr_2$ (0.005 g in 1 mL DMF), and Me_6TREN (0.01 mL).

For B_3M_7, Initiator (eBiB, 0.005 mL), monomer: MMA (7.52 g, 8.0 mL) and BA (9.79 g, 11 mL), solvents (anisole 8 mL, DMF 2 mL), $CuBr_2$ (0.005 g in 1 mL DMF), and Me_6TREN (0.01 mL).

Nuclear Magnetic Resonance Spectroscopy (NMR). Conversion of polymerization and the molar ratio of MMA/ BA in the product polymer were monitored by ^{1}H-NMR on a Bruker Advance 500 MHz NMR instrument in $CDCl_3$ at room temperature.

Procedures for fabrication of a bulk film. Brush particles and linear polymers were dispersed in THF via sonication and stirred for 24 h. Solution of brush particle/ linear polymer blends were made by mixing the solutions of brush particle and linear polymer by a certain volume ratio followed by stirring for 24 h. The bulk dispersions were transferred into 20 mm × 20 mm square Teflon molds or 15 mm × 5 mm rectangular Teflon molds. The solvent was slowly evaporated over 48 h at room temperature, generating transparent nanocomposite films with a thickness of 0.1-0.2 mm. The residual solvent was removed from the bulk films by transferring them to a vacuum oven set at 120 °C and annealed for 24 h.

Size Exclusion Chromatography (SEC). Number-average molecular weights (Mn) and molecular weight distributions of samples were determined by size exclusion chromatography (SEC). To perform SEC on brush particle samples, chains were cleaved from particles by etching of particles in HF in a polypropylene vial for 24 h, neutralized with ammonium hydroxide, and purified by passing through a column filled with basic alumina before running SEC. The SEC was conducted with an Agilent 1260 Iso pump and Waters 410 differential refractometer using PSS columns (Styragel 10^5, 10^3, 10^2 Å) with THF as an eluent at 35 ºC and at a flow rate of 1 mL min^{-1}. Linear PMMA standards were used for calibration. Toluene was used as internal standards for the testing.

Thermogravimetric Analysis (TGA). TGA with TA Instruments 2950 was used to measure the mass fraction of SiO_2 in the brush particles. The data were analyzed with TA Universal Analysis. The heating procedure involved four steps: (1) jump to 120 ºC; (2) hold at 120 ºC for 10 min; (3) ramp up at a rate of 20 ºC/min to 800 ºC; (4) hold for 5 min. Grafting density was calculated using formula (eq. 1).

$$\sigma_{TGA} = \frac{(1-f_{sio2})N_{Av}\,\rho_{sio2}\,d}{6\,f_{sio2}\,M_n} \qquad \text{(eq. 1)}$$

where f_{SiO2} is the SiO_2 fraction measured by TGA, N_{Av} is the Avogadro number, ρ_{SiO2} is the density of SiO_2 nanoparticles (2.2 g cm^{-3}), d is the average diameter of SiO_2 nanoparticles (113.2 nm), M_n is the overall number-average MW of the cleaved polymer brushes.

Transmission Electron Microscopy (TEM). Monolayered brush particle and blend films were obtained by dropping diluted solution (solvent: toluene) onto copper grids followed by annealing at 120 °C for 24 h to fully equilibrate. TEM was carried out using a FEI Tecnai F20 at 200 kV. The diameters and inter-particle distances of the SiO_2 nanoparticles were determined from statistical analysis of the TEM micrographs using MatLab software.

Differential Scanning Calorimetry (DSC). The glass transition temperature (T_g) of linear copolymers was measured by differential scanning calorimetry (DSC) with TA Instrument QA2000. The same procedure was run three times, each involving the following steps: (1) Equilibrate at 25.00 °C, (2) Isothermal for 1.00 min, (3) Ramp 20.00 °C/min to -90.00 °C, (4) Isothermal for 1.00 min, (5) Ramp 20.00 °C/min to 160.00 °C, (6) Isothermal for 1.00 min, (7) Ramp 20.00 °C/min to -90.00 °C, (8) Isothermal for 1.00 min, (9) Ramp 20.00 °C/min to 160.00 °C, (10) Isothermal for 1.00 min, (11) Ramp 20.00 °C/min to -90.00 °C, (12) Isothermal for 1.00 min, (13) Ramp 20.00 °C/min to 160.00 °C, (14) Isothermal for 1.00 min, (15) Jump to 25.00 °C. The DSC data were analyzed with a TA Universal Analysis instrument, and T_g was directly acquired.

Tensile test. the linear copolymer bulk films are tested in the tensile mode by using DMA (TA RSA-G2). The film thickness was between 150-200 μm. The samples were stretched at a constant tensile rate of 0.05 s^{-1} at room temperature.

Self-healing Test. *(A) Scratch healing.* A stainless-steel razor blade was used to cut a '#' shape (~20 μm in width, ~100 μm in depth, ~2 mm in length of each cut) on the bulk film surface at room temperature. The image of the film was immediately taken by optical microscope, and the film was annealed in an oven set at 70 °C for specific times. After that, a series of photos were taken at different timescales. *(B) Cut-and-adhere healing.* A bulk film was severed, then physically reattached within 1 min at 110 °C, and allowed to self-heal for a specific time under 70 °C. After that, the same film after healing was characterized by a tensile test as described above. Then, compared the pristine samples and calculated the recovery ratios.

Small angle neutron scattering (SANS). (A) *Sample preparation.* Brush particles and linear polymers were dispersed in THF via sonication and stirred for 24 h. Solution of brush particle/ linear polymer blends were made by mixing the solutions of brush particle and linear polymer by a certain volume ratio followed by stirring for 24 h. The bulk dispersions were transferred into 20 mm × 20 mm square Teflon molds. The solvent was slowly evaporated over 48 h at room temperature, generating transparent nanocomposite films with a thickness of 0.1-0.2 mm. The residual solvent was removed from the bulk films by transferring them to a vacuum oven set at 120 °C and annealed for 24 h. (B) *Data Reduction Process.* Small angle neutron scattering (SANS) experiments were performed using the General-Purpose Small-Angle Neutron Scattering (GP-SANS) beamline at the High Flux Isotope Reactor in Oak Ridge National Laboratory. The obtained SANS data were initially reduced with the air background at the beamline. The following data normalization and background subtraction were then performed using SASVIEW. Following the data collection and reduction at the beamline, the reduced data were normalized with the thickness of each sample (Blend-20%, B_3M_7+5% SiO_2-B_3M_7, and

B_3M_7 as shown in **Fig. 1c**). B_3M_7 is treated as a linear polymer background and is used for background subtraction from both Blend-20% and B_3M_7+5% SiO_2-B_3M_7 to reveal only the scattering effect from the SiO_2 core. B_3M_7+5% SiO_2-B_3M_7 is further normalized with the number density to reveal the form factor of a single SiO_2 core. Since the SANS results of Blend-20% contain both the structure factor and the form factor, the structure factor of Blend-20% can be obtained by eliminating the obtained form factor of SiO_2 core from B_3M_7+5% SiO_2-B_3M_7. The structure factor result is shown in **Figure 1(d)**.

Supporting data

Table S1. Characteristics of BA-*stat* -MMA copolymer blend systems.

Entry	*Tg (℃)*	*Tg Range (℃)*
10%B_5M_5	38	21~50
20% B_5M_5	35	17~51
30% B_5M_5	28	11~45
50% B_5M_5	21	4~38

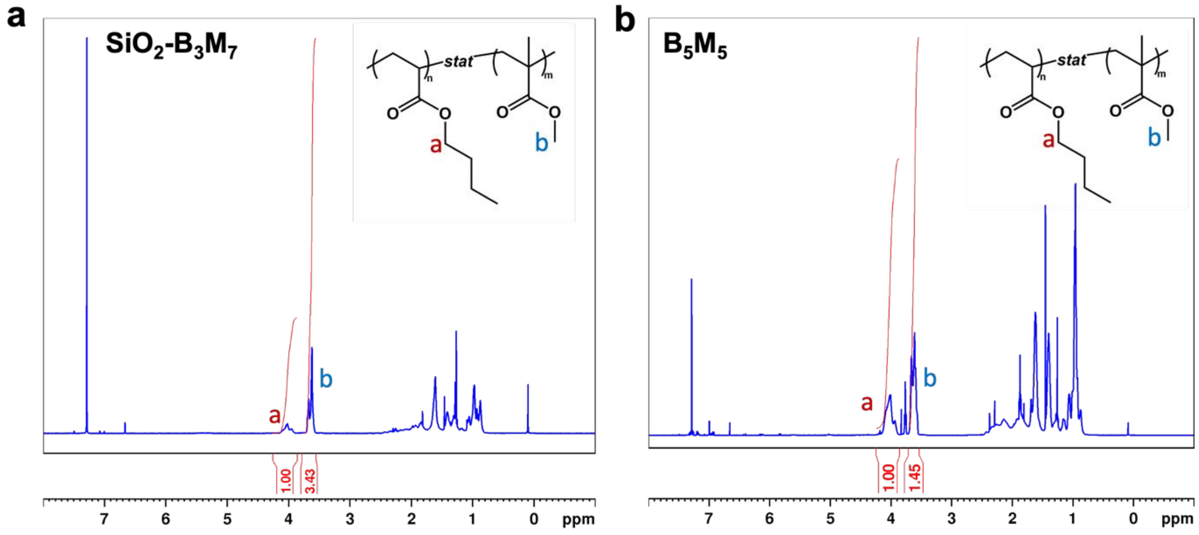

Figure S1. NMR results of (a) SiO_2-B_3M_7 and (b) B_5M_5.

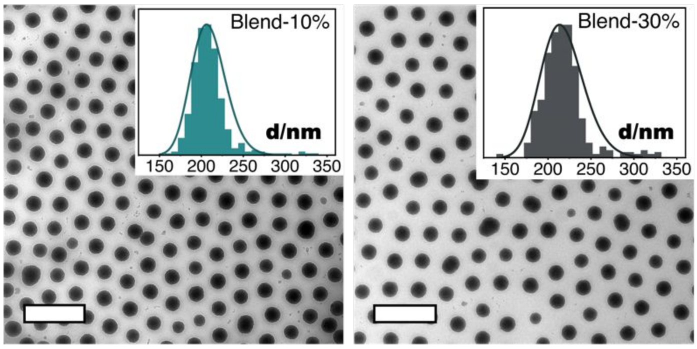

Figure S2. TEM images of (left) Blend-10% and (right) Blend-30%. Scale bar: 500nm. The insets show the distribution of interparticle distances calculated based on the TEM images.

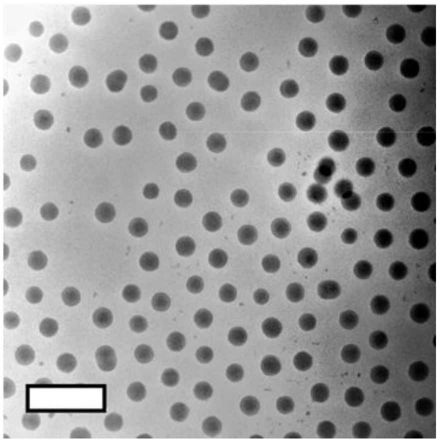

Figure S3. TEM images of Blend-50%. Scale bar: 500 nm.

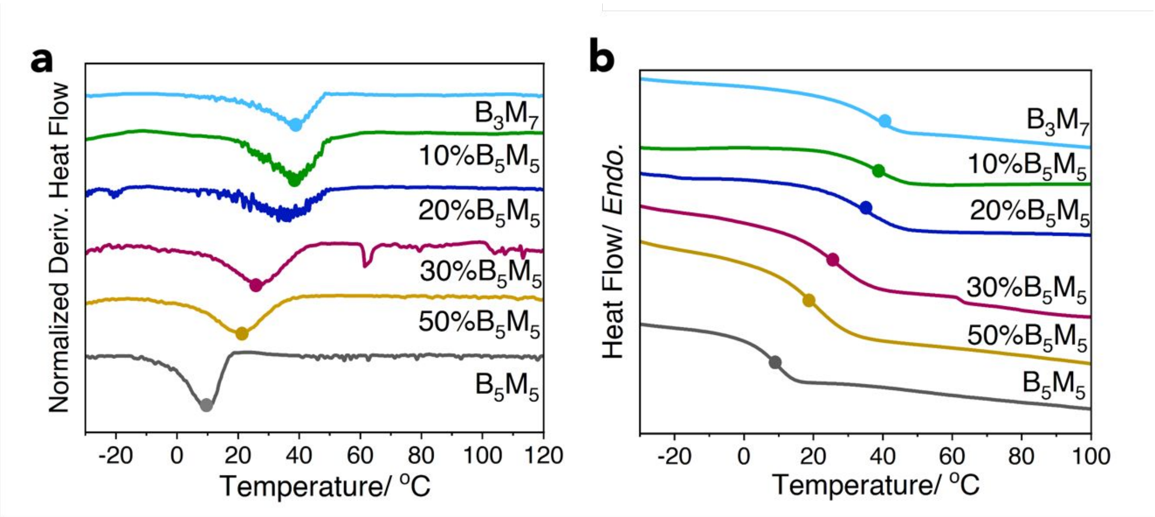

Figure S4. DSC results of B_5M_5/B_3M_7 linear blends. (a) Heat flow curves and (b) normalized derivative heat flow curve. The respective glass transition temperatures are highlighted with solid points. All curves were recorded during the 3rd heating/cooling run at a heating rate of 20 ℃ min^{-1}.

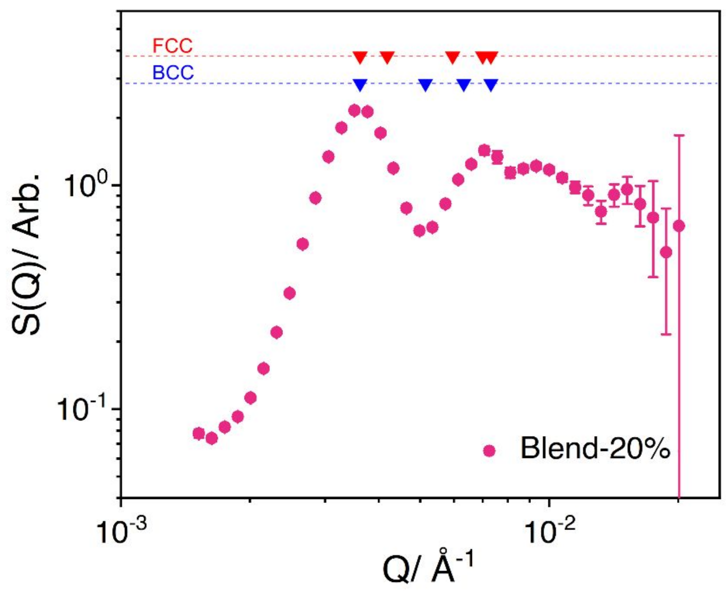

Figure S5. The structure factor results of Blend-20% with predicted FCC and BCC structure factor peaks assuming the first peak location corresponding to {110} and {111} reflections for BCC and FCC, respectively.

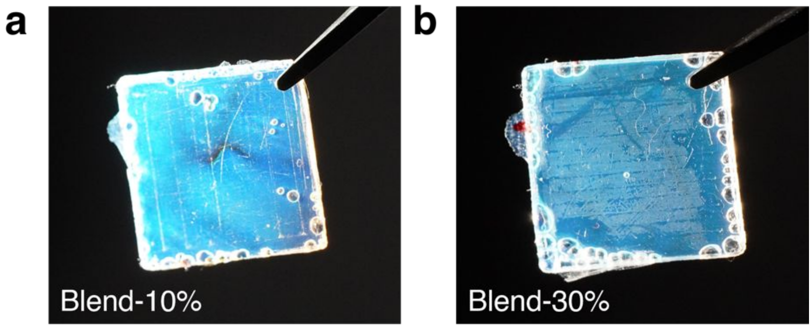

Figure S6. Photos showing (a) Blend-10% bulk film and (b) Blend-30% bulk film.

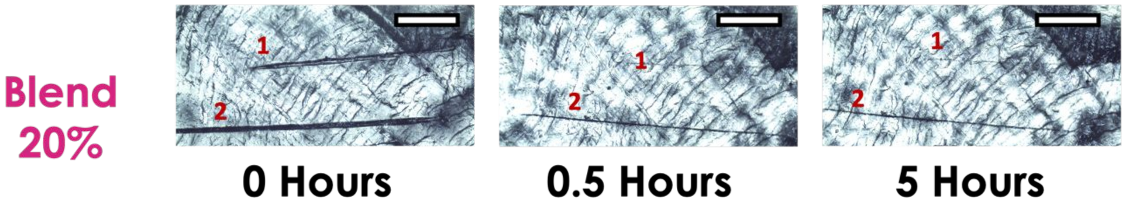

Figure S7. Stereomicroscopic images of the healing process for Blend-20% with different scratch depth. Top (scratch 1): depth approximately 50 μm. Bottom (scratch 2): depth approximately 100 μm. The scratch film was allowed to heal under 70 °C. Scale bars are 500 μm.

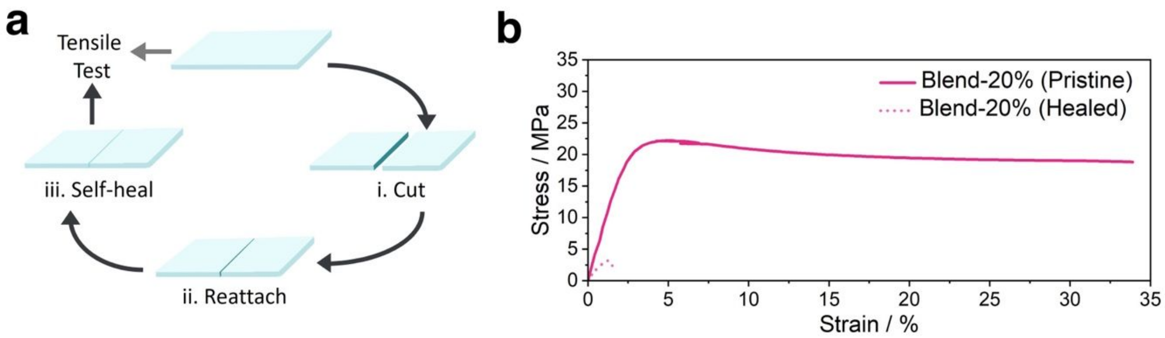

Figure S8. (a) Illustration of cut-and-adhere self-healing test. (b) Strain-stress curves for pristine and damaged-and-healed (healed at 70 ^{o}C) for films.

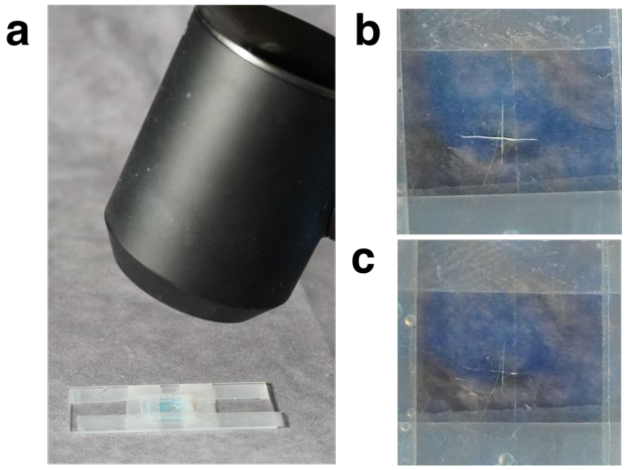

Figure S9. Scratch healing with hair dryer. (a) Experiment setup: the film was taped to a glass slide to prevent being blown away during the experiment. The hair dryer (Dyson) was set to high temperature and low wind power. (b) The film after scratching. (c) The film after being heated with the hair dryer for 3 mins.

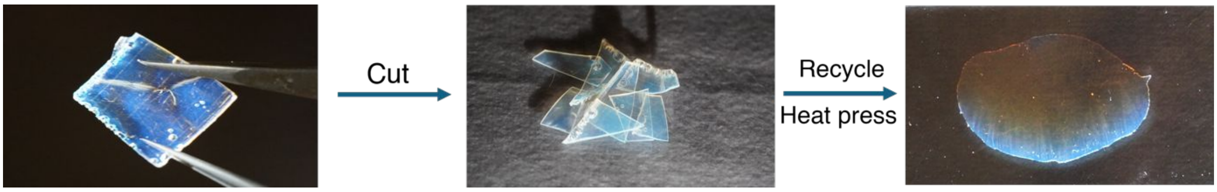

Figure S10. Re-processability of Blend-20%. The bulk film was cut into pieces with a razor and heat-pressed (70℃, 1000 psi) for 5 minutes.

Recording Files

Video S1 Bending process of bulk film of SiO_2-B_3M_7.

Video S2 Bending process of bulk film of Blend-20%.

Video S3 Shape transition by shape memory effect. Shape memory process for Blend-20% bulk film. The sample folded automatically from a 2D shape to a corresponding self-standing "flower" shape. Heat plate temperature is 70 ℃.
